# Understanding Retention in Pre-Exposure Prophylaxis Care in the South: Insights from an Academic HIV Prevention Clinic

**DOI:** 10.1089/aid.2021.0177

**Published:** 2022-04-06

**Authors:** Charles M. Burns, Monica Borges, Justin Frye, Kathryn Keicher, Scotty Elliott, Sheila Schwartz, Kenneth Shipp, Nwora Lance Okeke, Mehri S. McKellar

**Affiliations:** ^1^ Division of Infectious Diseases, Duke University Medical Center, Durham, North Carolina, USA.; ^2^ Duke Center for AIDS Research, Duke University, Durham, North Carolina, USA.; ^3^ Department of Case Management and Duke University Medical Center, Durham, North Carolina, USA.; ^4^ Department of Pharmacy, Duke University Medical Center, Durham, North Carolina, USA.

**Keywords:** HIV, pre-exposure prophylaxis, persistence in care, sexually transmitted infections

## Abstract

HIV pre-exposure prophylaxis (PrEP) is poorly utilized in the southern United States. We examined PrEP retention in care and sexually transmitted infections (STIs) through a retrospective review of the Duke University PrEP Clinic from January 1, 2015 to October 15, 2019. We evaluated short-term (3 months), long-term (additional 8–12 months), and longitudinal retention in care in our clinic. Adjusted odds ratios (aOR) were generated to explore demographics associated with retention. Kaplan–Meier curves were generated to view retention longitudinally. STIs were examined at baseline (1 year before initial PrEP visit) and while retained in care. Of a total of 255 patients; 88% were men, 37% were black, and 73% were men who have sex with men (MSM). Short- and long-term retention in care were met by 130/237 (55%) and 80/217 (37%) patients, respectively. MSM were more likely to be retained in the short term (aOR = 5.22, 95% confidence interval [CI] = 1.57–17.32). Self-referred patients were more likely to be retained in the long term (aOR = 2.18, 95% CI = 1.12–4.23). Uninsured patients were less likely to be retained in the long term (aOR = 0.32, 95% CI = 0.11–0.91). STI diagnoses include 42 infections at baseline and 69 infections during follow-up. STI diagnosed while in PrEP care was associated with longer retention in care over time. Patients discontinue PrEP care over time and STIs were frequently encountered. Additional studies are needed to determine the best way to retain patients in HIV preventative care.

## Introduction

HIV pre-exposure prophylaxis (PrEP) given as once daily emtricitabine/tenofovir disoproxil fumarate or emtricitabine/tenofovir alafenamide is one of the most effective tools in the prevention of HIV acquisition.^1–4^ The Centers for Disease Control (CDC) recommends that all patients receiving PrEP be seen every 3 months in follow-up to ensure they are HIV negative, to assess medication adherence and side effects, and to conduct sexually transmitted infection (STI) testing for sexually active persons with symptoms and asymptomatic men who have sex with men (MSM) at high risk.^5^ However, a limitation to PrEP use is that it must be taken daily with frequent outpatient follow-up.

Since the rollout of PrEP programs nationally, there has been growing interest in persistence in care in follow-up visits.^6–8^ Previous large studies in the United States have shown a wide range of retention rates in PrEP care.^9–12^ Few studies have focused on PrEP retention in the southern United States, which accounted for more than half of new HIV diagnoses in 2016 but was also the region with lowest PrEP use nationally.^13,14^ Early reports on the rate and timing of disengagement from PrEP care in the south indicate that retention is worse than other regions ranging between 32% and 63% over time.^8,15–17^ More focus is needed on persistence in care and predictors for disengagement to better design interventions.

In addition to HIV preventative services, PrEP clinics also provide STI care. STIs have been increasing in the United States with >2.5 million cases of gonorrhea, chlamydia, and syphilis reported by the CDC in 2019. This finding represents an increase in the rate of chlamydia, gonorrhea, and syphilis of 19%, 56%, and 74%, respectively, since 2015.^18^ Southern states are frequently reported to have the highest incidence of STIs with North Carolina showing increased rates of STIs from 2018 to 2019.^19,20^ Increased rates of STI are also prevalent in adolescents, an age group that is high risk for acquiring HIV.^21^ Prior studies have shown an association between increased STI incidence and PrEP use.^22,23^ Yet it is unclear how prevalent this association is in the southern United States.

In this study, we report on retention in care and STIs encountered in a large southern academic PrEP clinic in Durham, North Carolina, over a 4-year time period. Our aim was to describe short- and long-term retention in care and patient characteristics associated with retention in care. A secondary aim was to evaluate incident STI diagnoses, which serve as markers of HIV exposure, while in care.

## Methods and Methods

### Patient population

Data were reviewed from the Duke University PrEP Clinic, which was established in 2015. This academic hospital-associated clinic provides PrEP services for adult patients who are at high risk for HIV from a wide area in central North Carolina, which includes both urban and rural counties. The clinic is staffed by several providers with pharmacy and social work support who assist in acquiring access to the medication and provide psychosocial counseling. All patients who were seen within the Duke PrEP Clinic since inception in 2015 were eligible for inclusion in the study. Patients who had a diagnosis of HIV at time of first encounter in the Duke PrEP Clinic were excluded.

### Data collection

We conducted a retrospective chart review of eligible patients from January 1, 2015 to October 15, 2019. Clinical data, including age, race, ethnicity, gender, sexual practice, insurance status, and referral source, were obtained from the Duke institutional data warehouse and through manual chart review.

Retention in care was determined by manual review of completed in-person follow-up encounters after the initial visit. Short-term retention in care was defined as completion of a 3-month follow-up as per CDC guidelines.^5^ Long-term retention was defined as completion of a 3-month visit and an additional visit between 8 and 12 months after the initial encounter. Discontinuation of care was defined as a lack of follow-up visit for 6 months since the last encounter. Patients were excluded from further analysis after their first discontinuation of clinic care**.** These definitions were chosen to reflect a real-world experience in our clinic.

STI diagnoses were extracted from the medical records and included syphilis serologies, genital and extragenital chlamydia, and gonorrhea nucleic acid amplification testing, hepatitis B serologies, and diagnosis of giardiasis. Baseline STI was defined as a diagnosis at or within 1 year before the initial PrEP visit. STI diagnoses while on PrEP were any subsequent diagnosis while retained in care regardless of diagnosis location. We considered empiric treatment for an STI the same as a new diagnosis even if laboratory testing was not performed. If a patient had two infecting organisms diagnosed at the same time, they were considered to have two new incident STIs.

### Data analysis

Outcomes of interest were short- and long-term retention in PrEP care and STI acquisition while on PrEP. Multivariable logistic regression was conducted to explore associations between patient-level determinants and outcomes of interest (SAS 9.4, Cary, NC). Kaplan–Meier curves were generated, and the log-rank test was used to compare longitudinal retention in PrEP among different patient groups (R, 3.6.0). The Kaplan–Meier curves used discontinuation from clinic care (no PrEP Clinic visit for 6 months) as the event of interest. This study was approved by Duke University Institutional Review Board.

### Institutional approval

This study was approved by the Duke University Institutional Review Board (IRB Protocol 00103503).

## Results

A total of 255 patients attended at least one PrEP clinic encounter (Table 1). Of these patients, 227 (88%) were male, 95 (37%) were black, and 186 (73%) identified as MSM. Median age at first visit was 33 years with an interquartile range of 17 years. Overall there were 153/255 patients aged ≤35 years. The majority of patients reported 2–4 sexual partners in the preceding 3 months (*n* = 84, 33%). Nearly a quarter were self-referred (*n* = 62, 24%) with other referral sources, including medical providers (*n* = 91.36%), community organizations (*n* = 30, 12%), dating apps (*n* = 27, 11%), peers (*n* = 20, 8%), health departments (*n* = 9, 4%), insurance (*n* = 3, 1%), and unknown sources (*n* = 13, 5%). A total of 153 (60%) patients returned for at least one follow-up with first follow-up appointment ranging from 30 to >270 days past the initial PrEP encounter.

**Table 1.** Pre-Exposure Prophylaxis Clinic Patient Demographics

Demographic	*N* (%) (*N* = 255)
Age at initial PrEP visit	
17–25	63 (24)
26–35	90 (36)
36–45	51 (20)
46–55	37 (15)
≥56	14 (5)
Gender	
Male	224 (88)
Female	25 (10)
Transgender female	6 (2)
Race/ethnicity	
Black	95 (37)
White	122 (48)
Multiracial/other	21 (8)
Hispanic/Latino	14 (5)
Declined	17 (7)
Sexual practice	
MSM	186 (73)
HIV+ partner ever	76 (30)
Insurance status	
Uninsured	52 (20)
Referral source	
Medical provider	91 (36)
Self	62 (24)
Community organization	30 (12)
Dating apps	27 (11)
Peers	20 (8)
Health department	9 (4)
Insurance	3 (1)
Unknown	13 (5)

MSM, men who have sex with men; PrEP, pre-exposure prophylaxis.

**FIG. 1.**
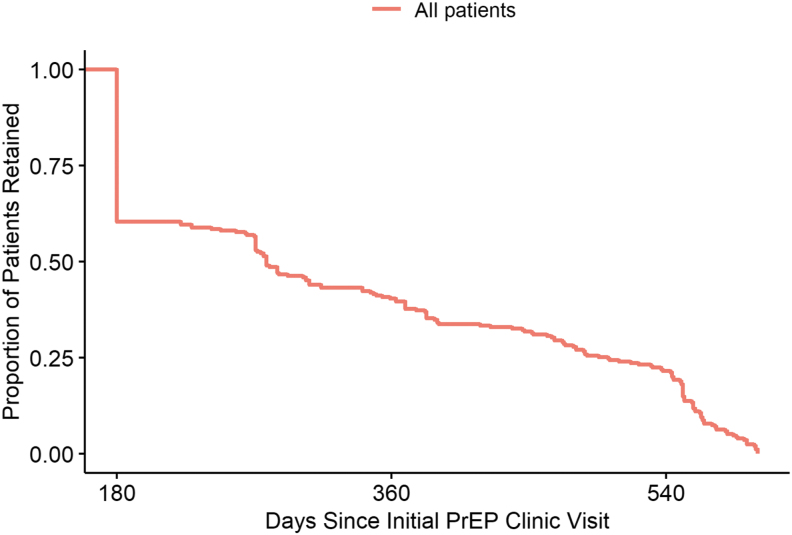
Overall retention in PrEP care for all patients. PrEP, pre-exposure prophylaxis.

Overall, our clinic patients frequently discontinue care over time with nearly all having their initial discontinuation of care within 1.5 years of starting PrEP (Fig. 1). Short- and long-term retention in care were met by 130/237 (55%) and 80/217 (37%) patients, respectively. MSM were more likely to be retained in the short term (adjusted odds ratio [aOR] = 5.22, 95% confidence interval [CI] = 1.57–17.32). Self-referred patients were more likely to be retained in the long term (aOR = 2.18, 95% CI = 1.12–4.23), whereas patients without insurance were less likely to be retained in the long term (aOR = 0.32, 95% CI = 0.11–0.91) (Table 2).

**Table 2.** Adjusted Odds Ratios for Short- and Long-Term Retention in Care at 3 and 8–12 Months, Respectively

Variable	Completion of 3-month appointment OR (95% CI)	Completion of 8–12 month appointment OR (95% CI)
Female	2.81 (0.73–10.8)	0.17 (0.01–1.48)
Black	0.81 (0.45–1.46)	0.83 (0.39–1.79)
Hispanic	1.42 (0.42–4.76)	0.96 (0.22–4.11)
MSM	**5.22 (1.57–17.32)**	1.46 (0.39–5.37)
Uninsured	0.50 (0.25–1.02)	**0.32 (0.11–0.91)**
Self-referred	1.18 (0.67–2.07)	**2.18 (1.12–4.23)**
HIV+ partner	0.89 (0.44–1.78)	1.66 (0.72–3.85)
35 years old and under	0.87 (0.50–1.52)	0.59 (0.30–1.13)
Baseline STI	0.81 (0.35–1.86)	1.95 (0.73–5.18)

Bold values are statistically significant.

CI, confidence interval; OR, odds ratio; STI, sexually transmitted infection.

**FIG. 2.**
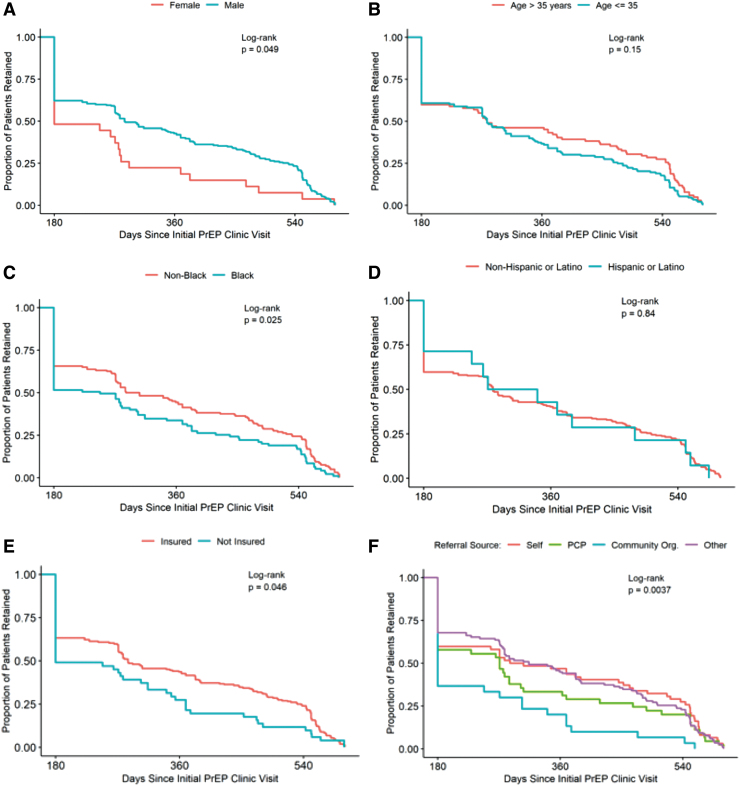
Retention in care over time based on patient demographics. **(A)** Retention by gender. **(B)** Retention by age. **(C)** Retention by race. **(D)** Retention by ethnicity. **(E)** Retention by insurance status. **(F)** Retention by referral source*. *Other referral sources include other medical providers, dating apps, peers, health departments, insurance, and unknown referral sources.

**FIG. 3.**
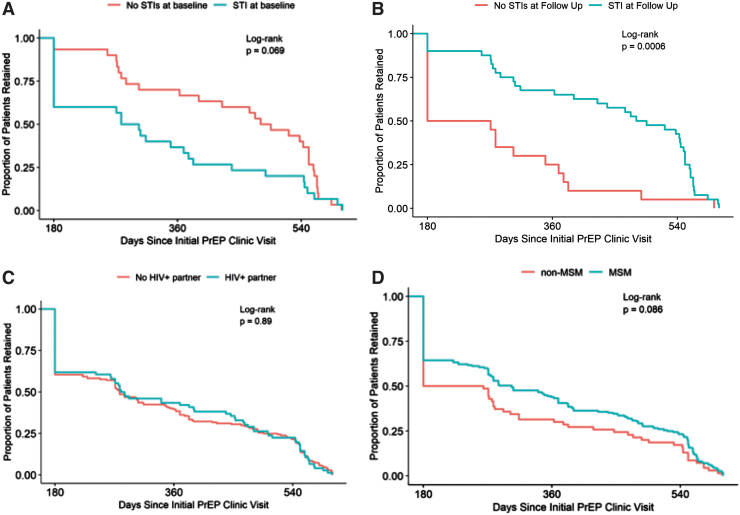
Retention in care over time based on sexual practice. **(A)** Retention with STI at baseline visit. **(B)** Retention with STI at follow-up visit. **(C)** Retention by HIV status of sexual partner. **(D)** Retention for MSM. MSM, men who have sex with men; STI, sexually transmitted infection.

We also examined retention until first discontinuation of care longitudinally in our clinic using Kaplan–Meier curves and the log-rank test (Figs. 2 and 3). Male patients remained in care longer than female patients (*p* = .049), but there was no statistical difference between age (greater or less than 35) (*p* = .15). Nonblack patients remained in care longer than black patients (*p* = .025), but there was no difference between ethnicity (Hispanic/Latino vs. Non-Hispanic/non-Latino) (*p* = .84). Patients with insurance remained in care longer than uninsured patients (*p* = .046). Retention was similar from different referral sources, including self and primary care physicians, except from community organizations, which was lower (*p* = .0037).

STIs diagnoses were made in 30 (12%) patients at baseline for a total of 42 unique infections. Of these baseline infections, 36% were syphilis, 32% were gonorrhea, 25% were chlamydia, 5% were *Giardia* and hepatitis B, and 2% were incident HIV infection. After initial PrEP visit, 44 (17%) patients had incident STIs detected for a total of 69 unique infections consisting of 6% syphilis, 38% gonorrhea, 55% chlamydia, and 1% *Giardia* and Hepatitis B combined. Two new HIV diagnoses were made at the initial PrEP encounter before starting PrEP medication.

No new HIV diagnoses were made during follow-up visits. Patients with an STI at baseline had no significant difference in persistence in care over time compared with patients without baseline STIs (*p* = .069). However, patients diagnosed with STIs during PrEP follow-up remained in care longer than those without a new STI diagnosis (*p* = .0006). There was no difference in retention between persons with or without an HIV-positive sexual partner (*p* = .89). Longitudinally, there was no significant difference in persistence in care between MSM and non-MSM when followed to first discontinuation of care (*p* = .086).

## Discussion

We present 4 years of data from a large academic PrEP clinic in the south. We focused on short term (3 months) and long term (an additional visit between 8 and 12 months) to reflect the CDC guidelines, previously published timeframes, and our clinical experience.^5,23–28^ Over time, patient retention in care declined with approximately half completing a 3-month follow-up visit and just more than one-third completing a longer-term visit 8–12 months from the initial visit. This is consistent with what has been previously reported in other southern PrEP clinics.^8,15,16,28^ However, persistence in PrEP care is generally worse in the south when compared with the Midwest and western United States.^6,27,29–32^

In our analysis, MSM and self-referral were predictive of persistence in care. Both groups may be more motivated to stay in care due to increased perceived risk for HIV and/or greater awareness and willingness to take PrEP among MSM and the fact that self-referred patients had sought out care on their own versus being referred by another provider or agency.^33,34^ When followed over time, nonblack patients and male patients were retained longer than black and female patients, respectively. Uninsured patients were less likely to remain in PrEP care in the long term. In our PrEP clinic, uninsured patients are eligible for financial assistance to alleviate the clinical care and laboratory costs associated with PrEP, and medication is obtained through pharmaceutical-sponsored drug assistance programs.

Therefore, patients likely face other socioeconomic barriers to remaining on PrEP care such as transportation or stable housing.^35–37^ It is notable that patients referred from community-based organizations appeared to be less apt to be retained in care than those referred from medical providers, dating apps, insurance providers, peers, self-referrals, health departments, insurance, and unknown referral sources. Community-based organizations are key partners to our clinic and often serve populations who are difficult to reach but would greatly benefit from HIV prevention efforts. Further collaboration with these organizations is needed to help increase PrEP use and retention.

Interestingly, we found no difference in retention from patients with or without an HIV-positive sexual partner. Having an HIV-positive sexual partner who is not virally suppressed is one of the key indications for PrEP use. The lack of a difference in retention among this group may be indicative of the awareness of the recent U = U campaign promoting that undetectable viral load means HIV is untransmittable.^38^ Perhaps with this recent breakthrough, persons on PrEP may reassess their risk for HIV acquisition and decide to stop the medication.

Finally, women were shown to fall out of care sooner than men. Although this may be due in part to the lower number of women in our clinic, it may also be an indication that we are not meeting the needs of female PrEP users. Women are a key group who need access to PrEP as they comprised 6,700 new HIV infections nationally, yet only 7% of eligible women were receiving PrEP in 2018.^39,40^ Similarly, we only had six transgender women in clinic during the time of our study and were unable to comment on trends in PrEP retention in this patient group. However, transgender women are an important group that are at risk for HIV acquisition and are in need of PrEP services.^41–43^ Further efforts are needed to engage this population in HIV preventative care.

Patients with baseline STI diagnoses were less likely to remain in care. Although there are many possible reasons for PrEP disengagement, having an STI has been identified as a reason for PrEP discontinuation in other cohorts.^44^ However, it is interesting that in our clinic having an STI diagnosis made while on PrEP was associated with retention in care. A similar finding was reported in an earlier retrospective study that included a portion of our clinic.^16^

Retention in PrEP care after an STI diagnosis is possibly due to patients having comfort with a longitudinal sexual health provider and recognizing their high-risk sexual behavior for HIV acquisition. It remains unclear if the prevalence of STIs truly reflects increased rates of infection among PrEP patients or is simply reflective of frequent testing of a high-risk population. Our findings support the need for frequent follow-up visits with STI testing.

Limitations to our study include having data only from a single clinic within a large academic medical center. Therefore, we cannot account for experiences at health departments, private clinics, or community-based clinics in our region that provide PrEP services and may have differing retention rates. Similarly, we cannot account for STI diagnoses occurring outside of our medical system, which may result in underreporting of STI rates in our clinic. Another limitation is that some PrEP users will return to care after stopping therapy. Our study did not include persons who returned to care after their first discontinuation of PrEP. In addition, some PrEP patients are referred back to their primary care providers after PrEP initiation and others may have transferred care to providers outside of our clinic resulting in a lower retention rate in our clinic.

## Conclusions

Overall, our clinic patients discontinue PrEP care frequently in both the short and long term. We found that MSM were more likely to remain in care in the short term compared with non-MSM patients, whereas self-referred patients were more likely to remain in care in the long term as compared with those referred from other sources. When followed over time, male, nonblack, patients with insurance coverage and patients with an STI occurring while on PrEP more often remained in care. Future studies will be needed to fully understand why patients discontinue PrEP care and to determine the best way to recruit, engage, and better retain patients in care. This is especially important in the southern United States where improved PrEP use and retention are critically needed to combat the national HIV epidemic.
